# Amplitude of low-frequency fluctuations in classical trigeminal neuralgia patients with purely paroxysmal and concomitant continuous pain

**DOI:** 10.3389/fpain.2025.1523845

**Published:** 2025-03-10

**Authors:** He Zhao, Shenghui Xie, Xueying Ma, Xue Bai, Yuanjun Song, Qiong Wu, Yang Gao

**Affiliations:** ^1^ Department of Radiology, The Affiliated Hospital of Inner Mongolia Medical University, Hohhot, Inner Mongolia Autonomous Region, China; ^2^ First Clinical Medical College, Inner Mongolia Medical University, Hohhot, Inner Mongolia, China

**Keywords:** classical trigeminal neuralgia, static amplitude of low-frequency fluctuation, dynamic amplitude of low-frequency fluctuation, magnetic resonance imaging, chronic pain

## Abstract

**Background:**

Purely paroxysmal neuralgia (PP-CTN) and concomitant continuous pain (CCP-CTN) are different subtypes of classical trigeminal neuralgia (CTN). Our aim was to explore the common and unique spontaneous brain activity abnormalities between subtypes.

**Methods:**

A total of 101 PP-CTN patients, 52 CCP-CTN patients, and 122 age- and sex-matched healthy controls (HCs) were included. All the subjects underwent resting-state functional magnetic resonance imaging, and changes in spontaneous brain activity were observed via whole-brain static amplitude of low-frequency fluctuation (sALFF) and dynamic amplitude of low-frequency fluctuation (dALFF).

**Results:**

Compared with HCs, PP-CTN patients presented significantly lower sALFF values in the left calcarine fissure and surrounding cortex (CAL), left putamen, and left Rolandic operculum (ROL). Compared with HCs, CCP-CTN patients presented significantly increased sALFF values in the left superior frontal gyrus (SFG), right medial superior frontal gyrus (MSFG), left putamen, right insula, and brainstem. Compared with the PP-CTN group, the CCP-CTN group presented significantly greater sALFF values in the left CAL, left SFG, right MSFG, left putamen, right insula, left ROL and brainstem. The results of the dALFF analysis revealed that, compared with HCs, PP-CTN patients presented increased dALFF values in the anterior cingulate gyrus (ACG) and decreased dALFF values in the right cuneus. Compared with HCs, CCP-CTN patients presented increased dALFF values in the ACG, right insula, and brainstem and decreased dALFF values in the right cuneus. Compared with the PP-CTN group, the CCP-CTN group presented increased dALFF values in the right insula and brainstem.

**Conclusions:**

Our findings reveal different neural mechanisms between PP-CTN and CCP-CTN patients, providing important neuroimaging evidence to better understand the pathophysiology of different subtypes of CTN.

## Introduction

Classical trigeminal neuralgia (CTN) is a chronic and debilitating pain disorder that significantly impacts patients' quality of life and daily functioning (1). The pathophysiologic mechanism of CTN is unclear. Neurovascular conflict was first proposed, wherein the trigeminal nerve has immediate contact with or is displaced by adjacent blood vessels, and vascular compression leads to focal demyelination of the entry zone of the trigeminal nerve root, hyperexcitability of the myelinated axons, and increased susceptibility to ectopic excitation, epithelial transmission, and high-frequency discharges (2). Neuroimaging studies have shown that patients with CTN also have altered functional activity in localized brain regions, involving abnormal functional activity in pain modulation, affective, and sensorimotor brain regions, suggesting that abnormal brain function likewise plays a role in the development of CTN (3).

The most common subtype of CTN is purely paroxysmal classical trigeminal neuralgia (PP-CTN), where the pain usually manifests itself as sudden, brief episodes that last from a few seconds to 1–2 min (4). In addition to paroxysmal pain, a significant proportion of patients with CTN (24%–49% of cases) experience persistent background pain, which is commonly described as aching, nagging, boring, or dull. There have been various definitions of this condition in the past, including atypical CTN or CTN type 2 (5, 6). The International Headache Society classification now defines this condition as classical trigeminal neuralgia with concomitant continuous pain (CCP-CTN) (7). The mechanisms of the two different subtypes of CTN may differ, and peripheral or central sensitization may contribute to the pathophysiology of CCP-CTN. Additionally, pain relief tends to be less effective in CCP-CTN patients than in those with PP-CTN following conventional treatments, such as sodium channel blockers or microvascular decompression (8, 9).

The amplitude of low-frequency fluctuation (ALFF) is a useful indicator of the intensity of activity in brain regions. Previous studies have assumed that the signal of resting-state functional magnetic resonance imaging (rs-fMRI) is static during the scanning process (10, 11). However, the brain is not a static system but rather a complex network with highly dynamic properties, and the use of the static amplitude of low-frequency fluctuation (sALFF) alone may ignore the temporal variability of brain activity. The dynamic amplitude of low-frequency fluctuation (dALFF), which is based on the sliding window technique, divides the entire time series into several small windows, with each window containing a certain number of time points. These windows slide along the timeline at certain steps, capturing the dynamic characteristics of brain activity over time. The dALFF is an indicator of the variance in the ALFF and is an effective method for exploring the dynamic function of the brain; it has been applied in studies of schizophrenia, generalized anxiety disorder, and Parkinson's disease (12–14).

Previous studies have given limited attention to changes in dALFF in CTN, and research specifically focusing on the CCP-CTN remains scarce. Researchers often combine CCP-CTN with CTN for overall analysis, overlooking the distinct patterns of abnormal brain activity unique to this subtype. Therefore, this study analyzed rs-fMRI data of the PP-CTN and CCP-CTN subtypes and explored the abnormal sALFF and dALFF values of the two subtypes, providing a new perspective for a deeper understanding of the neural mechanisms of different CTN subtypes.

## Methods

### Participants

We recruited 153 patients with CTN and 122 matched healthy controls (HCs) to participate in this study. All of the patients were recruited from January 2019 to December 2023 at the Affiliated Hospital of Inner Mongolia Medical University, China. CTN was diagnosed according to the 3rd edition of the International Classification of Headache Disorders (ICHD-3) (7) and included 13.1.1.1.1, CTN, purely paroxysmal, in 101 patients and 13.1.1.1.2, CTN with concomitant continuous pain, in 52 patients. The inclusion criteria for CTN patients were as follows: (1) no abnormal signals on routine T1WI and T2WI sequences, which were used to exclude space-occupying lesions such as epidermoid cysts; (2) Presence of neurovascular compression determined via three-dimensional fast imaging employing steady-state acquisition (3D-FIESTA) and time-of-flight magnetic resonance angiography (TOF-MRA); (3) absence of other pain disorders (e.g., fibromyalgia, chronic low back pain, migraine) or neurological conditions that could interfere with the diagnosis of CTN and (4) right-hand dominance. The exclusion criteria for CTN patients were as follows: (1) other chronic headache disorders; (2) secondary trigeminal neuralgia caused by herpes or multiple sclerosis; (3) other serious physical or mental illnesses; (4) alcohol or drug abuse; (5) previous invasive craniocerebral surgeries; and (6) contraindications to MRI examination.

The inclusion criteria for HCs were as follows: (1) age > 18 years; (2) right-hand dominance; (3) no neuropsychiatric disorders; and (4) no contraindications to MRI. The exclusion criteria for HCs were as follows: (1) metal implants in the body, particularly metallic fixed dentures; (2) alcohol or drug abuse; and (3) mental health issues or severe sleep disorders.

### Questionnaires and ratings

CTN patients underwent scale assessments prior to the same-day MRI scan, including (1) the visual analog scale (VAS), which consists of a sequence of numbers from 0 to 10, where 0 indicates no pain and 10 indicates the worst pain; (2) the short form of the McGill Pain Questionnaire (SF-MPQ), which assesses the type, intensity, and impact of pain; and (3) the Hamilton Anxiety Rating Scale (HAMA) and the Hamilton Depression Rating Scale (HAMD), which are used to assess the severity of anxiety and depressive symptoms. All study participants were right-handed according to self-reports. The study adhered to the Declaration of Helsinki and was approved by the Ethics Committee of Inner Mongolia Medical University Affiliated Hospital, China, with approval number WZ2024007. All the subjects signed an informed consent form before the study.

### Data acquisition

Data were acquired via a Siemens MAGNETOM Skyra 3.0 T MR scanner (MAGNETOM Skyra, Siemens Medical System, Erlangen, Germany) and a 20-channel combined head and neck coil. 3D-T1-weighted structural images were acquired via a gradient echo sequence (TR = 2,300 ms, TE = 2.3 ms, FOV = 240 mm^2^, matrix = 256 × 256, slice thickness = 0.9 mm, and slice number = 192). rs-fMRI data were acquired via an echo-planar imaging sequence (TR = 2,000 ms, TE = 30 ms, FOV = 240 mm^2^, matrix = 64 × 64, flip angle = 90°, slice thickness = 4 mm, gap = 0 mm, and slice number = 35). During scanning, the study subjects were asked to relax and remain awake with their eyes closed.

### Data preprocessing

Data processing was performed via the toolbox for Data Processing & Analysis of Brain Imaging (DPABI, http://rfmri.org/dpabi). Data from the first 10 time points were removed to eliminate the effects of the initial magnetic field signal stabilization process and the study subject's adaptation to the scanning environment. Then, slice timing and head motion were performed on the remaining 180 volumes of images. The data were screened with a translation or rotation parameter of less than ±2.0 mm or ±2.0°. For spatial standardization, individual fMRI data were aligned with T1 structural images, resegmented into voxels 3 × 3 × 3 mm^3^ in size and normalized to Montreal Neurological Institute (MNI) space. Next, a 6-mm isotropic Gaussian kernel was used for spatial smoothing. Finally, a regression of covariates was performed, including the regression of potential confounding effects characterized by white matter time series (5 CompCor noise components), cerebrospinal fluid time series (5 CompCor noise components), Friston-24 parameters, and linear trends within each functional run.

### Quality control assessment

Visual inspection was conducted before preprocessing, with a focus on identifying and removing data with larger motion or metal artifacts. Data quality control was carried out using the standard quality control pipeline in functional connectivity toolbox (CONN, https://www.conn-toolbox.org) (15). Data with head movements exceeding 2 mm or 2°, framewise displacement values greater than 0.5 mm, and global signal changes exceeding 3 standard deviations were excluded.

### ALFF analysis

The bandpass filter for sALFF was chosen to range from 0.01 to 0.10 Hz. The dALFF values were set to a hamming window with a time window length of 30 TR and a step size of 1 TR. The sALFF values were calculated within each moving window. The standard deviation (SD) of the sALFF plots for all the moving windows was subsequently calculated to measure the dynamic changes.

### Statistical analysis

SPSS software version 23.0 (IBM Corporation, Armonk, NY, United States) was used for the demographic and clinical data analyses. Two-sample t tests (for age, education level, pain duration, total intracranial volume, framewise displacement, VAS, SF-MPQ, HAMA, and HAMD scores) and chi-square tests (for sex and pain laterality) were used to analyze the differences between patients with CTN and HCs. Analysis of covariance (ANCOVA) was used to compare the differences in whole-brain sALFF/dALFF values at the voxel level between the PP-CTN, CCP-CTN and HC groups, with age, sex, education, total cranial volume and mean framewise displacement (FD) as covariates. When comparing the PP-CTN and CCP-CTN groups, HAMA and HAMD scores, along with the medication distribution, were included as covariates.

For sALFF analysis, family-wise error (FWE) correction was applied at a significance level of *p* < 0.05 to control for multiple comparisons. For dALFF analysis, results are reported at an uncorrected voxel-level threshold of *p* < 0.001 due to the exploratory nature of the analysis. The sALFF/dALFF values of the significantly different clusters were extracted for *post hoc* comparisons. For each cluster, we performed pairwise comparisons between the PP-CTN, CCP-CTN, and HC groups, resulting in 3 pairwise comparisons per cluster. If there are a total of N clusters, the total number of comparisons is *N* × 3. Bonferroni correction was applied separately for sALFF and dALFF analyses by multiplying the raw *p*-values by the total number of comparisons (21 for sALFF and 12 for dALFF). Significance was determined at *p* < 0.05 after correction.

In addition, we correlated the sALFF/dALFF values in these significantly different clusters with the scale scores of all participants, including those in the PP-CTN, CCP-CTN, and HC groups, via Pearson correlation analysis.

## Results

### Demographic and clinical characteristics

Among the initial 153 CTN patients, 34.5% were diagnosed with CCP-CTN, which was lower than the proportion of PP-CTN patients. After excluding patients with missing images or incomplete scale scores and performing visual inspection of the raw, preprocessed, and denoised functional data, as well as applying quality control, a total of 81 PP-CTN patients, 47 CCP-CTN patients, and 101 HCs were ultimately included in the analysis. There were no significant differences between the PP-CTN, CCP-CTN and HC groups in terms of age, sex, education level, framewise displacement or total intracranial volume. There were no statistically significant differences between the PP-CTN and CCP-CTN groups in terms of pain side or duration. Compared with PP-CTN patients, CCP-CTN patients had higher HAMA and HAMD scores. The detailed demographic data and scale scores for all participants are summarized in Table 1.

**Table 1:** Demographics and clinical characteristics of the participants.

Variables	PP-CTN patients	CCP-CTN patients	Healthy controls	F/*P*-value
Sex (male/female)	36/45	18/29	46/55	0.67
Age (years)	60.08 ± 12.02	62.79 ± 11.66	59.39 ± 10.57	0.35
Education (years)	9.74 ± 3.83	8.89 ± 3.57	8.90 ± 2.26	0.48
Pain laterality (left/right)	31/50	18/29	–	0.99
Pain duration (months)	43.63 ± 52.68	52.70 ± 45.52	–	0.36
Total intracranial volume (L)	1.45 ± 0.18	1.42 ± 0.20	1.47 ± 0.12	0.90
Framewise displacement (FD)	0.12 ± 0.05	0.13 ± 0.07	0.11 ± 0.03	0.89
VAS	96.52 ± 12.59	97.23 ± 11.26	–	0.78
SF-MPQ	10.16 ± 7.37	12.37 ± 4.64	–	0.12
HAMA	4.63 ± 3.53	6.60 ± 4.41	–	0.01
HAMD	3.60 ± 2.52	6.08 ± 3.22	–	0.02
Medication	Carbamazepine (62)/ Pregabalin (10)/None (9)	Carbamazepine (32)/ Pregabalin (13)/dolantin(1)/None (1)	–	0.03

PP-CTN, classical trigeminal neuralgia, purely paroxysmal; CCP-CTN, classical trigeminal neuralgia with concomitant continuous pain; VAS, visual analog scale; SF-MPQ, short form of the McGill Pain Questionnaire; HAMA, Hamilton Anxiety Rating Scale; HAMD, Hamilton Depression Rating Scale.

### ALFF changes

**Figure 1:**
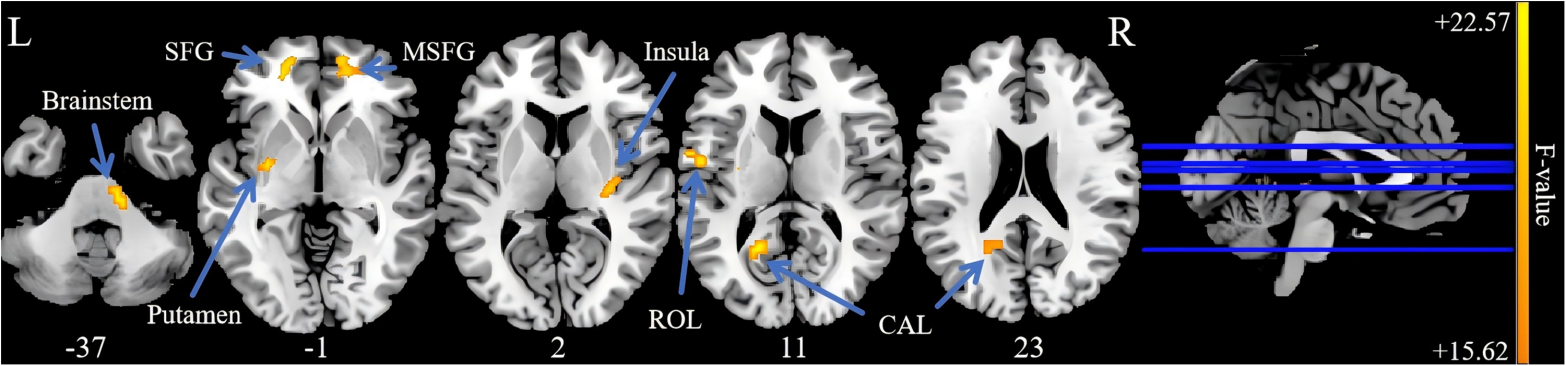
Significant differences of sALFF values among PP-CTN, CCP-CTN and HC (FWE correction). SFG, superior frontal gyrus; MSFG, medial superior frontal gyrus; ROL, rolandic operculum; CAL, calcarine fissure and surrounding cortex.

**Figure 2:**
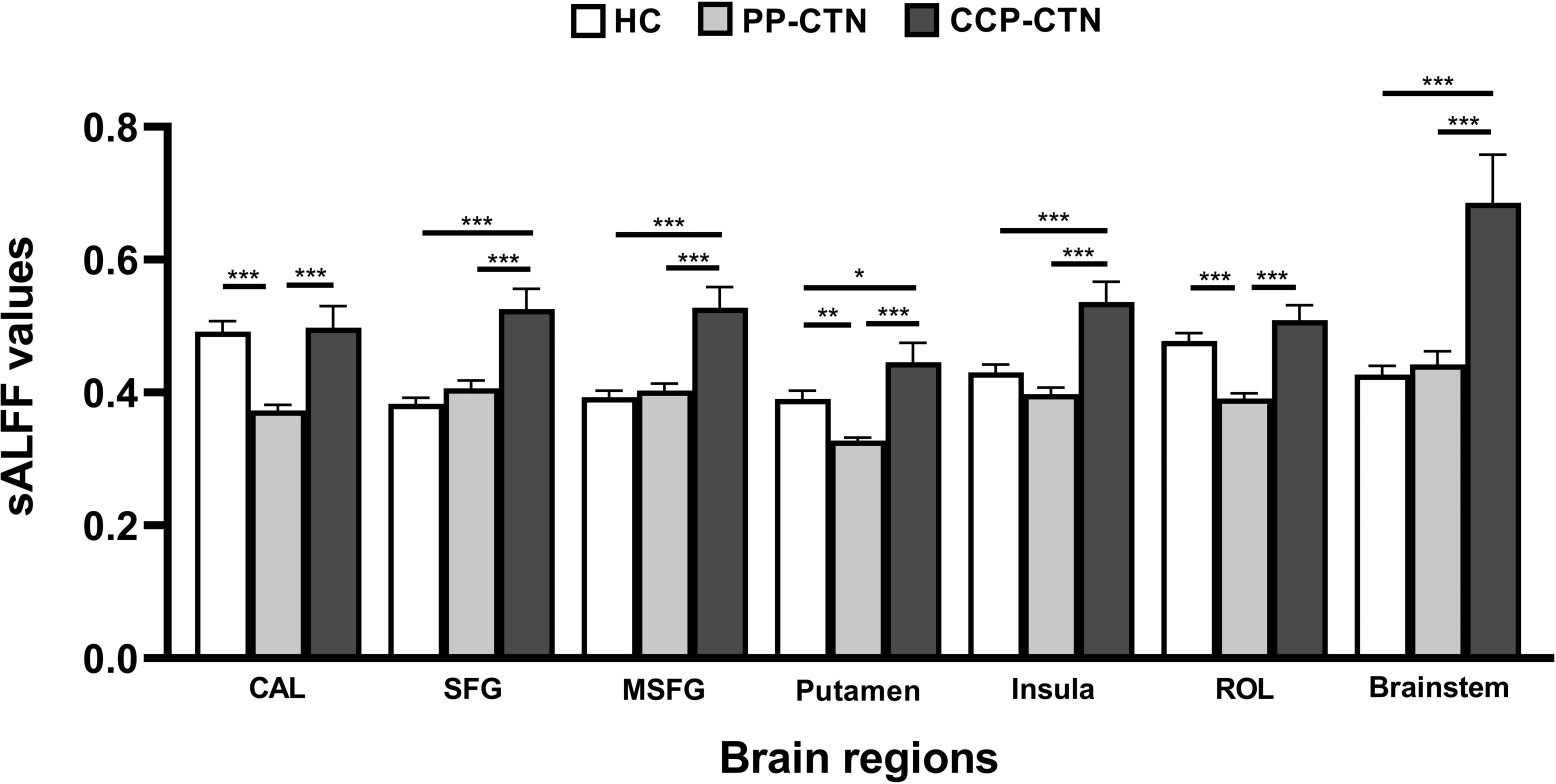
*Post hoc* comparisons of analysis of variance. The connection between two bars represents significant between-group differences (*represents significant level *p* < 0.05, **denotes significant level *p* < 0.01, and *** indicates significant level *p* < 0.001, Bonferroni correction). CAL, calcarine fissure and surrounding cortex; SFG, superior frontal gyrus; MSFG, medial superior frontal gyrus; ROL, rolandic operculum.

Three-group analysis of covariance revealed significant between-group differences in sALFF values in the left calcarine fissure and surrounding cortex (CAL), the left superior frontal gyrus (SFG), the right medial superior frontal gyrus (MSFG), the left putamen, the right insula, the left Rolandic operculum (ROL), and the brainstem (Table 2, Figure 1). Further intergroup analyses revealed significantly lower sALFF values in the left CAL, left putamen, and left ROL in the PP-CTN patients than in the HCs. Compared with HCs, CCP-CTN patients presented significantly increased sALFF values in the left SFG, right MSFG, left putamen, right insula, and brainstem. Compared with the PP-CTN group, the CCP-CTN group presented significantly greater sALFF values in the left CAL, left SFG, right MSFG, left putamen, right insula, left ROL and brainstem (Figure 2).

**Table 2:** Brain regions with differences in sALFF values among the three groups.

Regions	BA	AAL	Peak MNI			Cluster size	Peak *F* value
			X	Y	Z		
CAL	17	47	−18	−57	12	270	19.52
SFG	11	3	−21	53	−8	891	20.38
MSFG	10	20	12	54	3	1,053	18.17
Putamen	48	25	−26	−3	−5	1,036	13.74
Insula	48	34	33	−8	6	270	17.73
ROL	48	17	−54	−3	9	702	15.69
Brainstem	–	–	12,	−24	39	756	20.02

BA, Brodmann area; AAL, automated anatomical labeling atlas; MNI, Montreal Neurological Institute; CAL, calcarine fissure and surrounding cortex; SFG, superior frontal gyrus; MSFG, medial superior frontal gyrus; ROL, Rolandic operculum.

**Figure 3:**
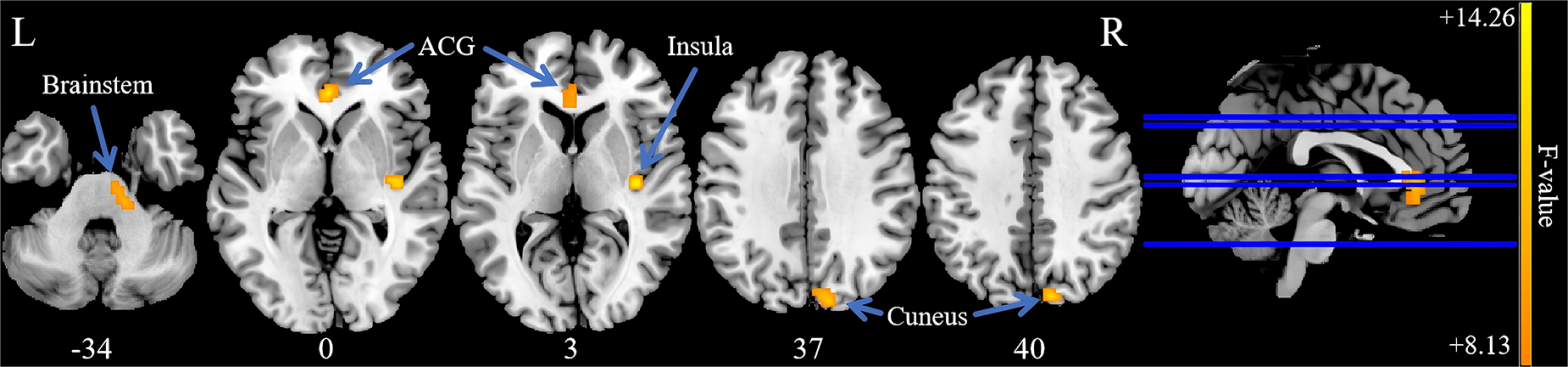
Significant differences of dALFF values among PP-CTN, CCP-CTN and HC. The results were obtained by ANCOVA analysis adjusted with age, sex, education, total intracranial volume and mean framewise displacement (*P* < 0.001, uncorrected). ACG, anterior cingulate gyrus.

**Figure 4:**
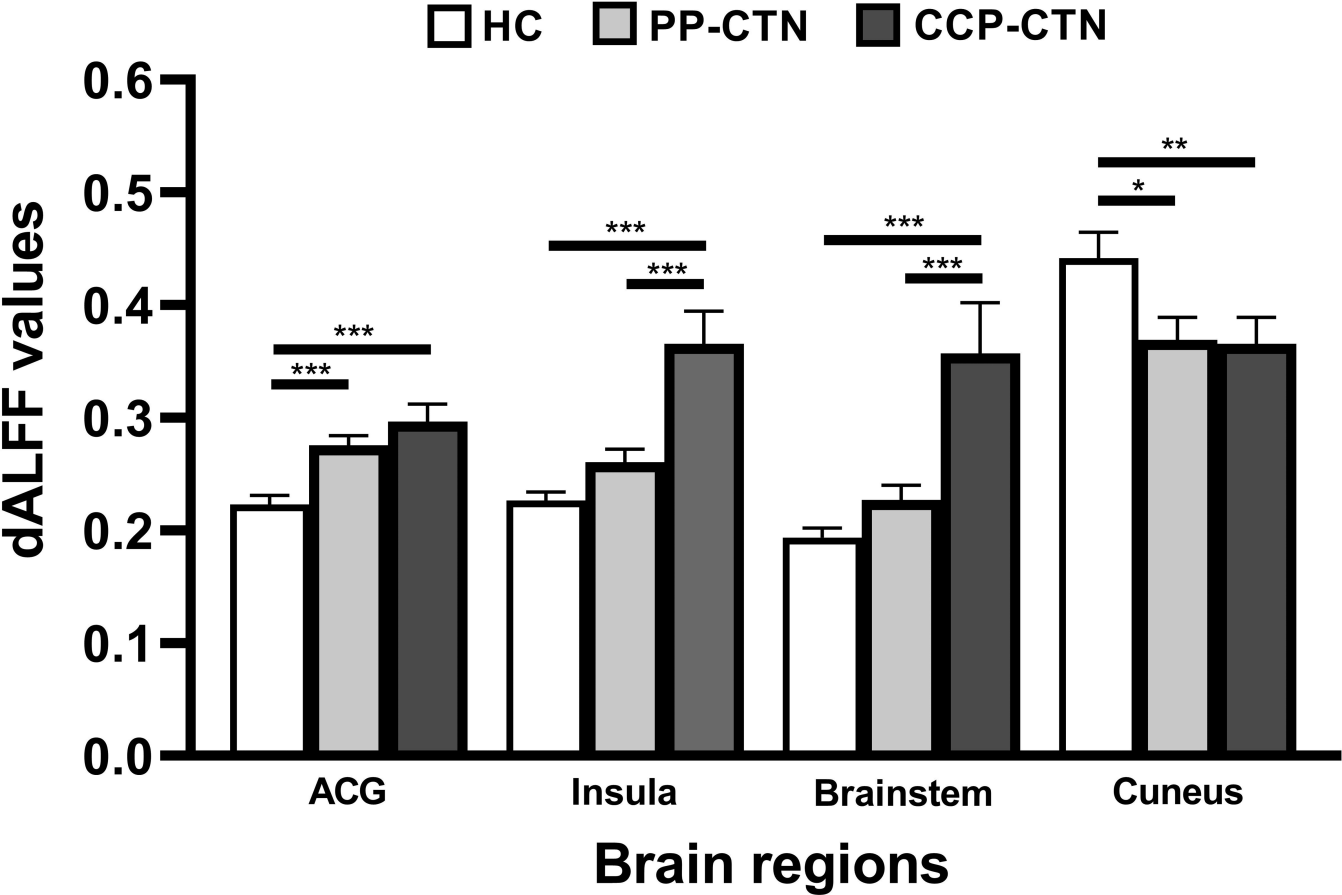
*Post hoc* comparisons of analysis of variance. The connection between two bars represents significant between-group differences (*represents significant level *p* < 0.05, **denotes significant level *p* < 0.01, and *** indicates significant level *p* < 0.001, Bonferroni correction). Abbreviations: ACG, anterior cingulate gyrus.

Analysis of variance of dALFF among the 3 groups revealed significant differences in dALFF in the anterior cingulate gyrus (ACG), right insula, brainstem, and right cuneus (Table 3, Figure 3). Further between-group analysis revealed increased dALFF values in the ACG and decreased dALFF values in the right cuneus in the PP-CTN patients compared with those in the HCs. Compared with HCs, CCP-CTN patients presented increased dALFF values in the ACG, right insula, and brainstem and decreased dALFF values in the right cuneus. Compared with the PP-CTN group, the CCP-CTN group presented increased dALFF values in the right insula and brainstem (Figure 4).

**Table 3:** Brain regions with differences in dALFF values among the three groups.

Regions	BA	AAL	Peak MNI			Cluster size	Peak *F* value
			X	Y	Z		
ACG	–	–	0	36	0	378	12.25
Insula	48	34	39	−18	3	297	9.04
Brainstem	–	–	12	−27	−42	796	11.77
Cuneus	19	50	12	−84	39	351	9.79

BA, Brodmann area; AAL, automated anatomical labeling atlas; MNI, Montreal Neurological Institute; ACG, anterior cingulate gyrus.

### Correlation with scale score tests

Group-based differences in the correlations between clinical characteristics and sALFF/dALFF values were observed in our research. However, no results were significant after multiple comparison corrections.

## Discussion

In the present study, we calculated the between-group differences in sALFF/dALFF values in the PP-CTN, CCP-CTN, and HC groups. Compared with those of HCs, the sALFF values in the left CAL, left putamen, and left ROL of PP-CTN patients were significantly lower. Compared with HCs, CCP-CTN patients presented significant increases in sALFF values in the left SFG, right MSFG, left putamen, right insula, and brainstem. Compared with HCs, PP-CTN patients presented increased dALFF values in the ACG and decreased dALFF values in the right cuneus. Compared with HCs, CCP-CTN patients presented increased dALFF values in the ACG, right insula, and brainstem and decreased dALFF values in the right cuneus. In addition, compared with those in PP-CTN patients, altered sALFF/dALFF values were detected in more nodes in patients with CCP-CTN. Specifically, CCP-CTN patients yielded significantly increased sALFF values in the left CAL, left SFG, right MSFG, left putamen, right insula, left ROL, and brainstem. Additionally, the CCP-CTN group presented increased dALFF values in the right insula and brainstem. Notably, both the right insula and brainstem showed changes in sALFF and dALFF values, and the CCP-CTN group presented higher sALFF and dALFF values than the PP-CTN or HC groups did. Our results indicate that the spontaneous changes in brain activity intensity between PP-CTN and CCP-CTN patients differ, reflecting the different neural mechanisms underlying the two CTN subtypes.

Previous studies have often reported changes in sALFF in pain matrices such as the ACG, basal ganglia, and insula in CTN patients (16, 17), whose functional changes are believed to be associated with pain-related emotions and cognition. However, not every study has shown abnormal activation of the entire pain matrix, which is related to individual differences in pain, different durations of pain, applicability to the brain, and dynamic changes in neural networks (18, 19). Our results indicate that, compared with those in HCs, the sALFF values of the left CAL, putamen, and ROL in PP-CTN patients were lower, and these changes suggested that neuronal activity in these brain regions was weakened. The CAL is located on the inner side of the occipital lobe and is related to visual information processing. Abnormal activation of the CAL has recently been reported in patients with pain after total knee arthroplasty and those with neuropathic pain after brachial plexus avulsion, indicating that the CAL may also be involved in pain information processing (20). The ROL is an important region of the brain that is responsible for emotional processing and regulation and is closely related to pain experience (21). A recent study supported the ROL as a potential target for noninvasive brain stimulation in the treatment of chronic pain (22). The putamen is a part of the basal nucleus of the brain involved in the emotional regulation of pain and the adjustment of pain thresholds (23).

Compared with HCs, CCP-CTN patients presented higher sALFF values in the left SFG, right MSFG, right insula, and brainstem. CCP-CTN patients with concomitant persistent pain had an increased number of brain regions with significantly elevated sALFF values and enhanced disruption, and persistent pain inputs may play a key role (24). Increased sALFF values may be associated with the pathophysiological mechanisms of concomitant persistent pain, reflecting a unique pattern of neural activity in concomitant persistent pain, culminating in a pathological pain network state (25); thus, further validation via functional network studies is warranted. The SFG is involved in the process of emotion generation, expression, and regulation, with important implications for mood disorders such as depression and anxiety, and influences pain perception by modulating cortico-subcortical and cortico-cortical pathways through the inhibition of injurious transmission systems (26). Therefore, the increased activity we observed in the SFG may be a common manifestation of abnormal pain and pain comorbidity, and its increased functional activity may be a compensatory change in the brain. Interestingly, the right insula and brainstem in the CCP-CTN group showed a similar trend of increased sALFF/dALFF values. This reflects the increased intensity of overall activity in these brain regions, which are more sensitive to external stimuli or internal regulation (27, 28). The results we observed are located at the primary trigeminal sensory nucleus and may therefore be related to aberrant activation of pain transmission pathways in patients with CCP-CTN (29). Indeed, a growing body of evidence from experimental animal studies suggests that chronic neuropathic pain is associated with the plasticity of structures within the brainstem pain modulatory system and that altered control of the dorsal horn or spinal trigeminal nucleus by brainstem pain modulatory circuits leads to reduced analgesia and increased sensitivity to afferent somatosensory stimuli (30). Although the abnormal changes in ALFF values observed in the brainstem of CTN patients are interesting, the mixed symptom lateralization in the cohort complicates the interpretation. To address this, a *post hoc* subgroup analysis based on pain lateralization could provide more insight into whether the observed brainstem differences are strictly ipsilateral to the pain. We plan to include this analysis in future research to better understand the relationship between pain lateralization and brainstem activity. The insula is a multidimensional integration site for pain and is associated with pain intensity, anticipation, and negative emotions such as anxiety and depression (31). The increase in sALFF values in the insula may reflect the multiple pain features and intense pain intensity experienced by CCP-CTN patients.

dALFF is a means of capturing dynamic brain activity patterns based on high temporal resolution (32). Excessive variability or overstabilization of brain transient activity may be an indication of altered brain function and pathological states. In the dALFF analysis, CTN patients had increased dALFF values in the ACG and decreased dALFF values in the right cuneus, in both the PP-CTN and the CCP-CTN groups. The cingulate gyrus has many functions, including emotion regulation, decision making, and behavior regulation, and is critical for emotional stability and behavioral adaptation in individuals (33, 34). The ACG plays an important role in pain regulation and pain-related mood disorders; aberrant activation of the ACG has been observed in a variety of chronic pain studies (18, 34), and presynaptic and postsynaptic long-term potentiation may be an important physiological basis for abnormal ACG function (34, 35). The cuneus combines somatosensory inputs and cognitive processes, and cuneus activation has been observed in pain stimulation experiments, suggesting that it is commonly involved in pain processing (36). The cuneus is part of the default mode network (DMN), a set of brain region networks that show synchronized activity in the resting state and are involved mainly in cognitive functions such as self-introspection, memory retrieval, emotion regulation, and self-awareness (37). Previous observations of reduced functional activity in the cuneus of CTN patients (38), combined with our findings that the functional activity of the cuneus was maintained at a low level and with low dynamic variability, reflect a reduced level of self-concern in patients with CTN and the need to maintain a higher level of alertness to cope with the sudden onset of pain.

### Limitations

There are several limitations in this study that should be stated. First, we failed to exclude the potential effects of antiepileptic drugs on brain function, which is an important confounding factor in this study. Second, the current analysis focused on altered functional activity in localized brain regions, and further analysis is needed to determine whether TN patients have large-scale brain network abnormalities. The limitations of some scanning parameters need to be explained, including relatively long TRs and relatively large voxel sizes, which increases the risk of partial volume effects in small structures and the risk of undersampling physiological noise. Finally, for the dALFF analysis, our collection time point was relatively short.

## Conclusion

In this study, we compared spontaneous brain activity among PP-CTN patients, CCP-CTN patients, and HCs by analyzing sALFF and dALFF values. Our results revealed distinct differences in brain activity patterns across these three groups. When comparing the PP-CTN and CCP-CTN groups directly, CCP-CTN patients showed more extensive alterations in both sALFF and dALFF values, suggesting a more widespread and pronounced reorganization of brain activity in response to chronic pain. These findings may reflect the underlying neural mechanisms of chronic persistent pain in these two subtypes of CTN.

## Data Availability

The raw data supporting the conclusions of this article will be made available by the authors, without undue reservation.
